# Supplementary material for: Novel Imine-Tethering Cationic Surfactants: Synthesis, Surface Activity, and Investigation of the Corrosion Mitigation Impact on Carbon Steel in Acidic Chloride Medium via Various Techniques
Source: Molecules. 2023 Jun 3;28(11):4540. doi: 10.3390/molecules28114540 (PMC10254240; doi:10.3390/molecules28114540)
Supplement: Supplementary file 1 [file molecules-28-04540-s001.zip › molecules-2411157-supplementary.pdf]

Supporting Information's

Novel Imine-Tethering Cationic Surfactants: Synthesis, Surface Activity, and Investigation of the Corrosion Mitigation Impact on Carbon Steel in Acidic Chloride Medium via Various Techniques

Hany M. Abd El-Lateef [1,2,*], Ahmed H. Tantawy [3,*], Kamal A. Soliman [3], Salah Eid [3,4] and Mohamed A. Abo-Riya [3]

[1] Chemistry Department, College of Science, King Faisal University, Al-Ahsa 31982, Saudi Arabia
[2] Chemistry Department, Faculty of Science, Sohag University, Sohag 82534, Egypt
[3] Chemistry Department, Faculty of Science, Benha University, Benha 13518, Egypt;
kamal.soliman@fsc.bu.edu.eg (K.A.S.); eedsalah@yahoo.com (S.E.);
mohamed.aborya@fsc.bu.edu.eg (M.A.A.-R.)
[4] Chemistry Department, College of Science and Arts, Jouf University, Alqurayat 77455, Saudi Arabia
* Correspondence: hmahmed@kfu.edu.sa or hany_shubra@science.sohag.edu.eg (H.M.A.E.-L.);
ahmed.tantawy@fsc.bu.edu.eg (A.H.T.)

Contents

Title	Page
Materials	**3**
**Figure S1. IR, 1H, and 13C NMR spectra of ((2-chlorobenzylidene)amino)-N,N-dimethyl-N-(2-oxo-2-(decyloxy)ethyl) propan-1- ammonium chloride [ICS-10].**	**4**
**Table S1:** The effects of the addition of various concentrations of ICS-10 and ICS-14 on the weight of carbon steel in 1.0 M HCl solution.	**6**
**Table S2:** The inhibition capacity comparisons for some conventional Surfactants	**7**

Materials

The metal used is carbon steel (C-Steel) which has the following structure (wt./wt.%): 0.05 Ni, 0.02 Cr, 0.0256 Si, 1.81 Mn, 0.09 P, 0.1 C, 0.001 V, 0.01 Mo, 0.03 Cu, and the remainder is iron.

3-(*N, N* Dimethylamino)-1-propylamine (99%), 2-Chlorobenzaldehyde (97%), and Decyl (98%) were purchased from Acros organics Company (Belgium). Hexadecyl (97%) alcohol was attained from M/s S.D. Fine chemicals Pvt. Ltd (India) and tetrahydrofuran (99%) were obtained from Alnasr-chemical Company. Solvents (ethyl acetate, ethyl alcohol absolute (99%), and diethyl ether (99%) were obtained from Algomhoria Chemical Co., Cairo, Egypt. All the utilized solvents and reagents were received without further purification.

Figure S1. IR, ^{1}H, and ^{13}C NMR spectra of *((2-chlorobenzylidene)amino)-N,N-dimethyl-N-(2-oxo-2-(decyloxy)ethyl) propan-1- ammonium chloride* [ICS-10].

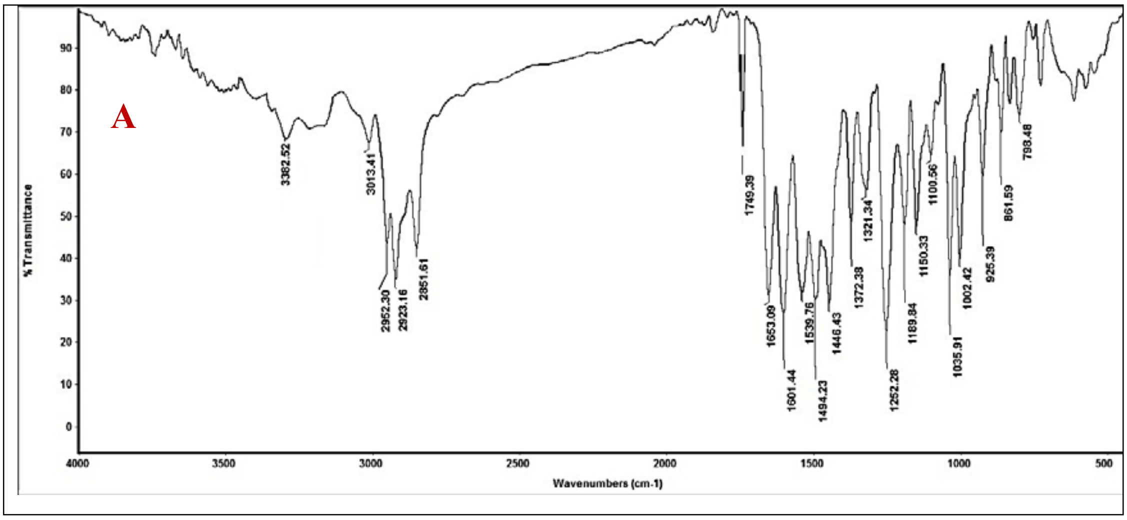

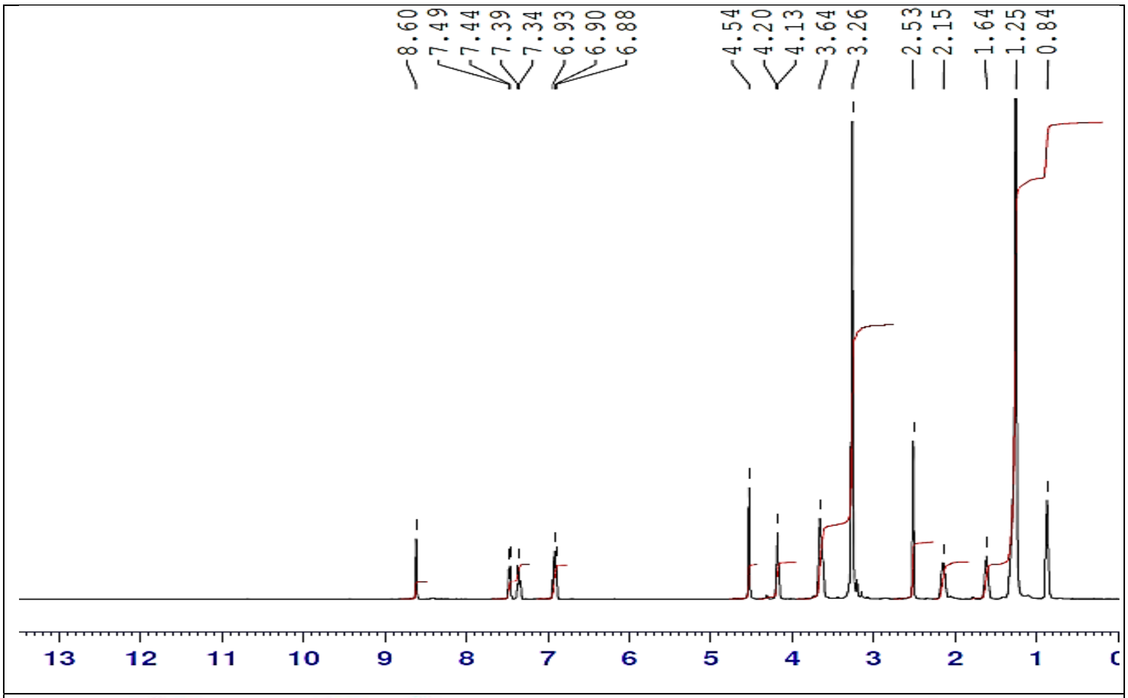
8.60
7.49
7.44
7.39
7.34
6.93
6.90
6.88
4.54
4.20
4.13
3.64
3.26
2.53
2.15
1.64
1.25
0.84
13 12 11 10 9 8 7 6 5 4 3 2 1

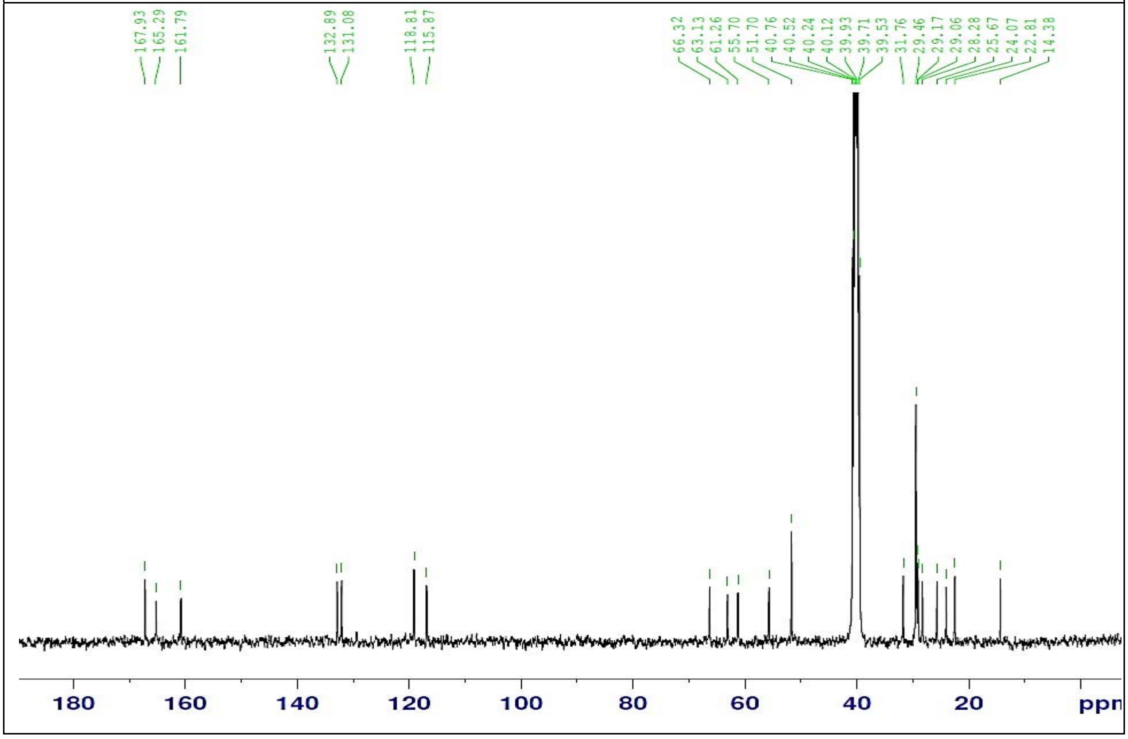
167.93
165.29
161.79
132.89
131.08
118.81
115.87
66.32
63.13
61.26
55.70
51.70
40.76
40.52
40.24
40.12
39.93
39.71
39.53
31.76
29.46
29.17
29.06
28.28
25.67
24.07
22.81
14.38
180 160 140 120 100 80 60 40 20

Table S1: The effects of the addition of various concentrations of ICS-10 and ICS-14 on the weight of carbon steel in 1.0 M HCl solution.

**Inhibitor code**	**Inh. Conc. (M)**	**Weight loss (gm)**	***θ***	***I.E/.%***
**-**	0	0.137	-	-
**ICS-10**	5×10^{-6}	0.070	0.4891	48.91
	1×10^{-5}	0.049	0.6423	64.23
	5×10^{-5}	0.027	0.8029	80.29
	1×10^{-4}	0.019	0.860	86.00
	5×10^{-4}	0.012	0.9113	91.13
**ICS-14**	5×10^{-6}	0.053	0.6131	61.31
	1×10^{-5}	0.040	0.7080	70.80
	5×10^{-5}	0.018	0.8686	86.86
	1×10^{-4}	0.014	0.8978	89.78
	5×10^{-4}	0.008	0.9416	94.16

Table S2: The inhibition capacity comparisons for some conventional Surfactants

**Inhibitor type**	**Corrosive medium**	**Inhibitor concentrat i on**	**Substrat e type**	**Inhibitio n capacity / %**	**Referen ce**
**ICS-10**	1 M HCl	0.5 mM	C- steel	~92	**Current study**
**ICS-14**	1 M HCl	0.5 mM	C- steel	~ 95	**Current study**
4-Diethyl Amino Benzaldehyde Schiff base cationic	2N HCl	200 ppm	C- steel	~94	**[1]**
Cetyl trimethyl ammonium bromide	0.5 M HCl	200 ppm	Mild-steel	~ 86.5	**[2]**
dodecyl trimethyl ammonium chloride	0.5 M HCl	200 ppm	Mild-steel	~ 87.1	**[2]**
3-((2-hydroxybenzylidene)amino)-N,N-dimethyl-N-(2-oxo-2-(decyloxy)ethyl) propan-1-ammonium chloride	3.5%NaCl +0.5 M HCl	150 ppm	Carbon Steel	~ 88.88	**[3]**
3-((2-hydroxybenzylidene)amino)-N,N-dimethyl-N-(2-oxo-2-(tetradecyloxy) ethyl) propan-1-ammonium chloride	3.5%NaCl +0.5 M HCl	150 ppm	Carbon Steel	~ 93.80	**[3]**
Quaternary ammonium surfactants	1.0 M HCl	720 ppm	C-steel	~ 85.0	**[4]**

References

1 Negm, N.A.; Zaki, M.F.; Salem, M.A.I. Synthesis and Evaluation of 4-Diethyl Amino Benzaldehyde Schiff Base Cationic Amphiphiles as Corrosion Inhibitors for Carbon Steel in Different Acidic Media. *J. Surfact. Deterg*. **2009**, *12*, 321–329. https://doi.org/10.1007/s11743-009-1156-0.

2 Fouda, A.S.; Elewady, Y.A.; Abd El-Aziz, H.K.; Ahmed, A.M. Corrosion Inhibition of Carbon Steel in 0.5 M HCl Solution Using Cationic Surfactants. *Int. J. Electrochem. Sci.* **2012**, *7*, 10456–10475. http://www.electrochemsci.org/papers/vol7/71110456.pdf.

3 Abd El-Lateef, H.M.; Tantawy, A.H. Synthesis and evaluation of novel series of Schiff base cationic surfactants as corrosion inhibitors for carbon steel in acidic/chloride media: Experimental and theoretical investigations. *RSC Adv*. **2016**, *6*, 8681–8700. https://doi.org/10.1039/C5RA21626E.

4 Hamitouche, H.; Khelifa, A.; Kouache, A.; Moulay, S. Petroleum quaternary ammonium surfactants mixture synthesized from light naphtha as corrosion inhibitors for carbon steel in 1 m HCl. *Corros. Rev*. **2013**, *31*, 61. https://doi.org/10.1515/corrrev-2012-0022.
